# Developmental Imaging of Radish Sprouts Using Dynamic Optical Coherence Tomography

**DOI:** 10.1002/jbio.202400254

**Published:** 2024-11-05

**Authors:** Yiheng Lim, Shumpei Kojima, Pradipta Mukherjee, Ibrahim Abd El‐Sadek, Shuichi Makita, Yoshiaki Yasuno

**Affiliations:** ^1^ Computational Optics Group University of Tsukuba Tsukuba Japan; ^2^ Centre for Biomedical Engineering Indian Institute of Technology Delhi Delhi India; ^3^ Department of Physics Damietta University Damietta Egypt

**Keywords:** botany, dynamic optical coherence tomography, optical coherence tomography, plant germination, plant imaging

## Abstract

The germination process of radish sprouts was investigated in detail using volumetric dynamic optical coherence tomography (OCT). Dynamic OCT involves the sequential acquisition of 16 OCT images and subsequent temporal variance analysis of each pixel, enabling non‐invasive visualization of the cellular and tissue activities of plants. The radish sprouts were longitudinally investigated for up to 12 days, and changes in morphology and dynamic OCT image patterns were observed as the plants developed. The dynamic OCT signals in the vessels and growing roots were relatively high in the early stage of germination and decreased as the tissue matured. These results suggest that dynamic OCT is sensitive to water and nutrient transport as well as cellular activities associated with plant growth.

## Graphical abstract

We non‐invasively investigated the germination process of radish sprouts for up to 12 days using volumetric dynamic optical coherence tomography (OCT). This technique analyzes temporal fluctuation in the OCT signal. The dynamic OCT signals in the vessels and growing roots were initially high and decreased with tissue maturation. These results suggest that dynamic OCT is sensitive to water and nutrient transport as well as cellular activities associated with plant growth.

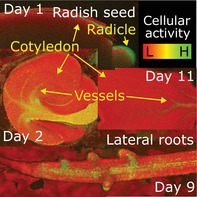

**Abbreviations**
D‐OCT: dynamic optical coherence tomography
LIV: logarithmic intensity variance
OCT: optical coherence tomography

## 1 Introduction

Understanding how different plant species develop is crucial for improving agricultural productivity and sustainability. Since plant development involves continuous changes in anatomy and physiology at a microscopic scale, understanding these changes requires the examination of living plants. A few model plants, such as *Arabidopsis thaliana*, *Oryza sativa*, and *Zea mays* have been extensively studied, but these models may not represent all plants [1]. Therefore, it is important to study a wider variety of living species.

Optical microscopy techniques are essential for characterizing living plants. Bright‐field microscopy was the first methodology used for observation of plant cells in Hooke's famous Micrographia [2] and is still widely used for plant investigation. Confocal microscopy has enabled optical depth sectioning by rejecting out‐of‐focus information and has improved the image contrast of optical microscopy. As a result, confocal microscopy is one of the most prevalent plant imaging modalities [3]. However, its imaging depth is restricted to a few hundred micrometers, limiting its application to thick plants [4].

The development of fluorophores and fluorescence microscopy has further advanced the study of plant physiology by enabling specific molecules to be labeled [5]. In particular, genetically encoded fluorescent proteins allow the non‐invasive, long‐term developmental study of living plants without the introduction of exogenous fluorophores [6]. For instance, cell nuclei can be labeled with fluorescent proteins for time‐lapse observation of cell division in living roots [7]. Although this modality is powerful, fluorescence microscopy based on genetically encoded fluorescent proteins is not applicable to non‐transgenic plants.

Optical coherence tomography (OCT) is a non‐invasive imaging technique for visualizing biological structures at micrometer‐scale resolution [8]. OCT can visualize plant anatomy at depths of up to 1 mm without sectioning and has been applied to many plant species. As examples, in vivo OCT was used to study the cellular structures of organs and seed germination of *Arabidopsis* [9]; germination of pea seeds [10]; root growth of switchgrass [11]; and thickness and refractive index of *Arabidopsis* leaves [12]. In addition, anatomical changes in diseased persimmon leaves [13], diseased cucumber [14] and melon seeds [15], and textural changes in maple leaves during senescence were studied ex vivo using OCT [16].

Tissue functions also can be investigated using dynamic OCT (D‐OCT), which visualizes cellular activities by analyzing the temporal fluctuation of the OCT signal over a period of a few seconds [17, 18]. To date, D‐OCT has been primarily applied to animals. For example, Caujolle et al. [19] measured the cellular motions and assessed the viability of bovine embryos; Apelian et al. [20] and Münter et al. [21] discriminated several cell types by analyzing the fluctuation rate of OCT signals in ex vivo rodent organs; Apelian et al. [20] visualized the alteration of rat liver metabolism induced by a metabolic inhibitor; Mukherjee et al. [22] visualized the time‐dependent degradation of dissected mouse liver by analyzing the magnitude of OCT signal fluctuations; El‐Sadek et al. [23, 24] quantified the metabolic and necrotic activities of human cancer spheroids and their drug response; Scholler et al. [25] visualized the organelle motility in retinal organoids generated from human induced pluripotent stem cells (hiPSCs); Morishita et al. [26] examined the intratissue and intracellular activities of hiPSCs‐derived alveolar organoids under normal and fibrosis conditions; and Hao et al. [27] mapped the spatial variation of heartbeats in hiPSCs‐derived heart organoids.

The temporal fluctuation of the OCT signal might also be used to characterize cellular activities in plants. However, in contrast to the wide application of D‐OCT to animals, it has rarely been used to study plant physiology. Among the few examples, Rajagopalan et al. [28] and Li et al. [29] used D‐OCT to measure the responses of leaves and seeds to chemical exposure. Similar to D‐OCT, laser speckle measurement was also used for monitoring plants. For instance, plant growth was investigated using point‐detection laser speckle measurements [30]. Additionally, en face imaging of vessels has been successfully performed [31, 32]. However, laser speckle measurement lacks depth resolution, which limits its ability to precisely identify the location of tissue activity.

In this study, we performed extensive physiological imaging on radish sprouts to demonstrate the potential of D‐OCT for characterizing cellular activity in plants. In contrast to the limited previous studies, the cellular activities in roots, hypocotyls, and leaves of the same sprouts were comprehensively investigated in vivo at different developmental stages, from seed germination to sprouting.

## 2 Materials and Methods

### 2.1 Samples and Protocol

**FIGURE 1:**
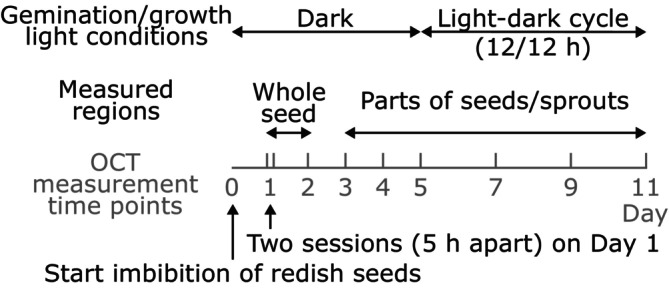
Timeline of the germination and optical coherence tomography (OCT) measurements of radish seeds. The seeds were imbibed on Day 0, and D‐OCT measurements were performed up to Day 11. The seeds were grown in the dark for the first 5 days and then exposed to a 12/12 h light–dark cycle. The whole seeds were imaged on Days 1 and 2, and parts of seeds or sprouts were measured on the other days.

Seven radish seeds (*Raphanus sativus* L. cv. Kaiwaredaikon), bought from a local grocery store, were germinated and grown on a petri dish at 20°C in a temperature‐controlled chamber for 12 days, as shown by the timeline in Figure 1. Imbibition was initiated on Day 0, and the seeds and radicles of their sprouts were kept moist with purified water and paper. For the first 5 days, the germination proceeded in darkness, and then the seeds were exposed to white light‐emitting diodes in a light–dark cycle of 12/12 h. After imbibition, the outer and inner cotyledons from each seed were grown, with one covering another. Because the cotyledons spread apart and adopt indistinguishable positions and appearances during sprouting, we loosely tied string around the petiole of one cotyledon to enable their discrimination.

Each seed and its growing sprout was scanned at several time points using a custom‐built swept‐source OCT system (see Section 2.2 for details). On Day 1, two sessions of OCT measurement were performed 5 h apart to image the fast‐emerging radicles. Each seed was imaged once per day from Days 2 to 5 and every 2 days thereafter (Figure 1). During each measurement session, a color photograph was acquired in addition to OCT images.

On Days 1 and 2, whole seeds were imaged. From Day 3, as the seeds germinated, OCT imaging of whole sprouts became impractically time‐consuming; hence, the cotyledons, hypocotyls, and radicles were individually imaged. Only the outer cotyledon of each seed was imaged from its adaxial side. In the early post‐imbibition days, one cotyledon covered another, and hence only the adaxial side of the outer cotyledon was measurable.

Prior to measurement, each seed or sprout was placed in a petri dish. By transversely moving the dish, we measured multiple overlapping transverse fields, which were then manually registered and stitched together to create a wide‐field composite image. For each field, the OCT probe head was adjusted axially to align the depth position of image.

### 2.2 OCT and D‐OCT

We used a custom‐built swept‐source Jones‐matrix OCT system, details of which are described elsewhere [33, 34]. Briefly, the wavelength of the light source sweeps around a central value of 1.3 μm at a scan rate of 50 kHz. The system has axial and lateral resolutions in tissue of 14.1 and 18.1 μm, respectively. The imaging depth range is 2.9 mm, and the probe power incident on the sample is 14 mW.

OCT imaging was performed using a repeating raster scan protocol with a field of view of 5 mm × 2 mm or 5 mm × 5 mm (fast direction × slow direction). The scanning range in the slow direction was determined by the size of the sample. The repeating raster scan protocol is compatible with volumetric D‐OCT imaging [24]. In the current implementation of this scanning protocol, the transverse field was split into four sub‐fields along the slow scan direction, and each field was raster‐scanned 16 times. Hence, a time sequence of 16 cross‐sections was obtained at each scan location, and each cross‐section consisted of 512 A‐lines. The inter‐frame time interval was 409.6 ms, and each sub‐field consisted of 32 slow‐scan locations, resulting in 128 slow‐scan locations and a total acquisition time of 26.2 s per volume. The number of cross‐sections and the total acquisition time at each scan location were optimized for the image contrast of tissue dynamics imaging previously [23].

Although the Jones‐matrix OCT device is polarization‐sensitive, we used only the polarization‐insensitive intensity image, which is obtained by averaging the squared image intensities of the four polarization channels [23]. The D‐OCT image was then computed from the 16‐frame time sequence of the polarization‐insensitive intensity images. We computed the variance of the time‐dependent OCT intensity in dB, known as the logarithmic intensity variance (LIV), to provide D‐OCT image contrast [23]. In addition, mean OCT intensity of the sequential 16 cross‐sections was computed. The mean was computed in linear scale, and then converted into dB scale. For better visualization, a pseudo‐color LIV image was composed by assigning the LIV and mean OCT intensity to the pixel hue and brightness, respectively. Using this colormap, low LIV appears as red, while high LIV appears as green.

En face slab projections of intensity and LIV images were generated by averaging the OCT intensity in linear scale and LIV across specific depth ranges below the surface of the samples. The sample surface was segmented from linear‐scale OCT cross‐sections. First, each cross‐sectional OCT image was denoised using a two‐dimensional Gaussian filter with a kernel size of 21 pixels × 21 pixels and a standard deviation of 2.5 pixels. Next, the denoised images were differentiated along the depth using a 3 pixels × 3 pixels Sobel operator, which calculates the derivative in the depth direction while applying a local weighted average in the transversal direction. The topmost pixels with differential values above a threshold were designated as the surface. It should be noted that in en face regions without a sample, the detected surface likely corresponds to the paper placed beneath the sample.

The superficial and deep slabs of the cotyledon were set at two specific depth ranges. The deep depth range was selected to consistently cover the veins at all time points, ensuring that the same tissue was imaged throughout the study. The intensity images are displayed in a logarithmic scale. Pseudo‐color en face projections of LIV were created by assigning the slab‐averaged LIV as the pixel hue and the OCT intensity as the pixel brightness.

## 3 Results

The radicles of all seven seeds emerged on Day 1. The hypocotyls emerged on Day 2 for six of seven seeds and on Day 3 for one seed.

In this section, we present representative images of two samples (denoted Samples 1 and 2). For Sample 1, images acquired on Days 1, 2, 5, 9, and 11 are presented. The results for Sample 2 are provided as an example of functional imaging of the root growth process.

### 3.1 Longitudinal Observation of Sample 1

#### 3.1.1 Photographic Observation

**FIGURE 2:**
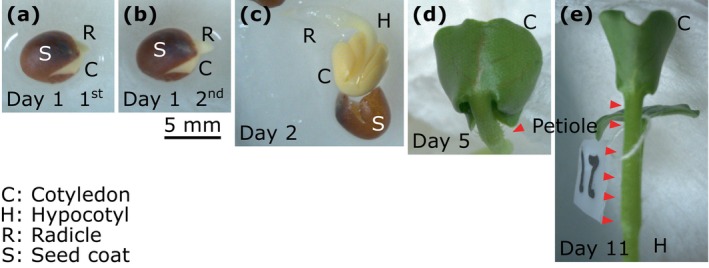
Photographs of Sample 1 on representative days after imbibition. (a, b) By Day 1, the seed coat (S) ruptured, and the cotyledon (C) and radicle (R) had emerged. (c) By Day 2, the seed coat had completely detached, and the hypocotyl (H) was visible. (d, e) On Days 5 and 11, the hypocotyl and cotyledon were green, and the petiole (red arrowheads) was developed. The string marker was used to identify the outer cotyledon.

Figure 2 summarizes the color photographs of Sample 1. In the first measurement session on Day 1 (Figure 2a), the seed coat ruptured, and the cotyledon and the radicle (an embryonic root) were visible. Five hours later, in the second imaging session on Day 1, we observed that the radicle had grown visibly larger (Figure 2b). By Day 2 (Figure 2c), both the outer and inner cotyledons had emerged from the seed coat and the hypocotyl was visible, with the cotyledons and radicle positioned either side. The cotyledons and hypocotyls appeared yellow because the seed was germinated in darkness, and the radicle was white. On Day 5 (Figure 2d) and Day 11 (Figure 2e), both cotyledons and the hypocotyl were green, and the petioles were developed between the cotyledons and the hypocotyl.

The outer cotyledon was folded and covered the inner cotyledon by Day 5, and both cotyledons had spread to opposite sides of the petiole by Day 11. Vascular structures were visible on the surface of the cotyledon on both Days 5 and 11.

#### 3.1.2 Day 1: Cotyledon and Emerging Radicle

**FIGURE 3:**
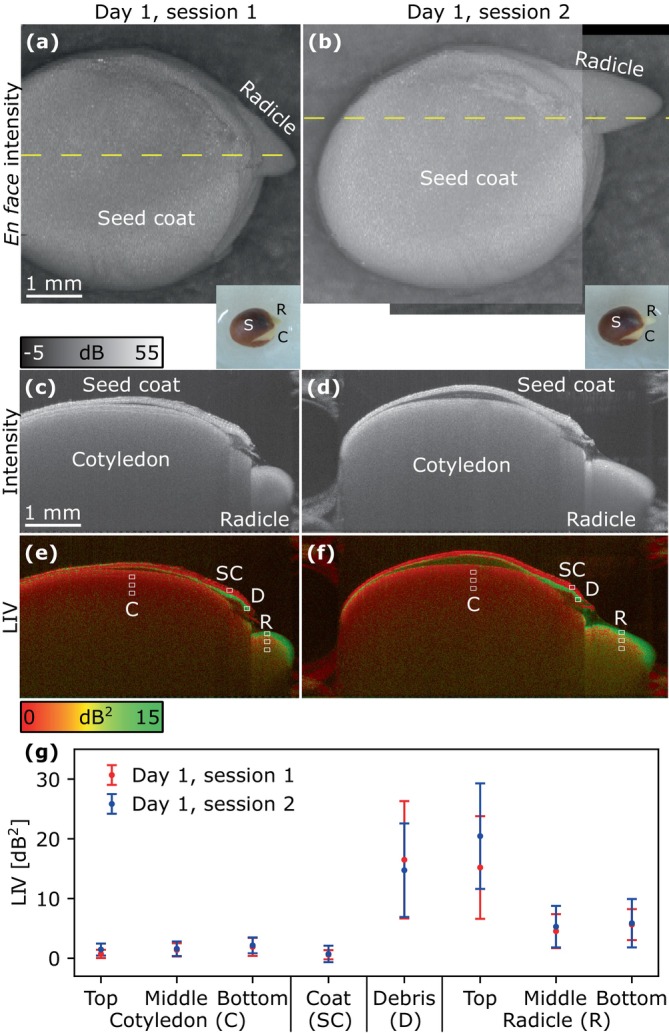
OCT and logarithmic intensity variance (LIV) images of Sample 1 on Day 1. Imaging session 1 (left column) was performed 5 h before session 2 (right column). (a, b) The first‐row images show full‐depth OCT projections, and (c, d) the second and (e, f) third rows show cross‐sections of the OCT intensity and LIV, respectively. The cross‐sections were obtained along the yellow dashed lines in the en face projections. The superficial region of the radicle exhibited a high LIV, which may indicate high activity of the newly emerging radicle. The inset color photographs in panels (a, b) are reproduced from Figure 2. The images obtained in imaging session 2 are wide‐field composites. The plot in panel (g) is the mean and standard deviation values of LIV for the ROIs within cotyledon (C), seed coat (SC), debris (D), and radicle (R) of (e, f).

The OCT analysis of Sample 1 on Day 1 is summarized in Figure 3, which shows the en face projection of OCT intensity images (Figure 3a,b), representative cross‐sectional OCT images (Figure 3c,d), the corresponding cross‐sectional LIV images (Figure 3e,f), and the mean and standard deviation values for the regions of interest (ROIs) within the LIV images (Figure 3g). The ROIs used in all studies are 10 × 10 pixels. The en face projection is an average of a thick slab that approximately contains almost all sample signals.

The OCT intensity images demonstrate the growth of the radicle over a period of 5 h. The cross‐sectional OCT images (Figure 3c,d) clearly captured the detachment of the seed coat from the cotyledon. In the LIV cross‐sections (Figure 3e,f), the cotyledon exhibited a low LIV (red), and the LIV slightly increased with depth (Figure 3g). In contrast, the superficial region of the radicle clearly exhibited a high LIV (green), which may indicate high activity of the newly emerging radicle. The outer part of the seed coat showed a low LIV, while the inner part exhibited a high LIV that may have originated from floating cell debris caused by the emergence of the radicle.

The cotyledon and seed coat exhibited the lowest LIV, followed by the radicle and cell debris (Figure 3g). The superficial (the top ROI) radicle had the highest LIV among the tissues. While LIV increased in the superficial radicle between the two measurement sessions, the changes in LIV for the cotyledon, inside (the middle and bottom ROIs) radicle, and seed coat were relatively small.

#### 3.1.3 Day 2: Growth of the Cotyledon, Hypocotyl, and Radicle

**FIGURE 4:**
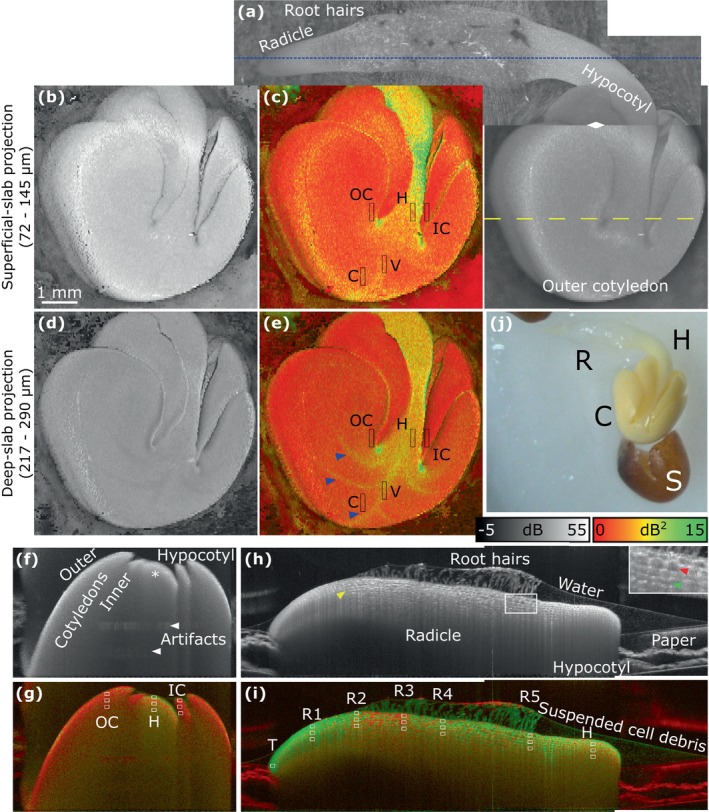
OCT intensity (grayscale) and LIV (pseudo‐color) images of Sample 1 on Day 2. (a) Wide‐field composite image of full‐depth OCT projections. Root hairs are visible on the radicle. The rhombus symbol indicates the line at which images measured using different axial positions of the OCT probe were stitched. (b, c) Superficial‐ and (d, e) deep‐slab projections. Blue arrowheads in panel (e) indicate the vessel structures which show a high LIV. (f, g) Cross‐sections along the yellow dashed line in panel (a), showing the hypocotyl (indicated by *) and the folded structure of the cotyledon. (h, i) Cross‐sections obtained along the blue dotted line in panel (a), which contain the hypocotyl and several zones of the radicle. ROIs in the LIV images were manually selected to calculate the mean and standard deviation values in Figure 5. The cell size increased along the length of the radicle and the yellow arrowhead indicates a large cell in the zone of the radicle without root hairs. In panel (h), the inset shows a magnified view of the region enclosed by the white rectangle beneath the root hairs, and the red and green arrowheads indicate large cells in superficial and deep layers, respectively. (j) Color photograph reproduced from Figure 2c.

Figure 4 summarizes the OCT and LIV images acquired on Day 2. Figure 4a is a wide‐field composite of OCT projections covering the whole sample, and the corresponding color photograph is shown in Figure 4j.

The wide‐field composite OCT image clearly revealed the structures of the cotyledon, hypocotyl, and radicle and also showed that the root hairs were visibly developed.

To investigate the cotyledon activity, OCT and LIV image projections were made at two depths (Figure 4b–e) in addition to the cross‐sectional images being obtained (Figure 4f–i). In the cross‐sectional OCT image (Figure 4f), the structures of the hypocotyl and inner and outer cotyledons were visible. The corresponding LIV cross‐section (Figure 4g) shows that the cotyledons exhibited a higher LIV (mixture of green and red) on Day 2 compared with that on Day 1 (Figure 3e,f), which may indicate that the corresponding tissue activity of the cotyledons was also higher. The hypocotyl also exhibited a high LIV (green).

In the superficial slab (72–145 μm below the surface), the LIV projection (Figure 4c) indicated that the hypocotyl had a high activity (green), whereas the inner and outer cotyledons had low (red) and relatively high (yellow) activities, respectively. Such a clear difference was not seen in the corresponding OCT projection (Figure 4b). In the deep slab (217–290 μm below the surface), high‐LIV vein structures were visible (Figure 4e), and these exhibited hypo‐scattering in the corresponding OCT projection (Figure 4d).

The root hair that was visible in the wide‐field OCT projection (Figure 4a) could be investigated in detail in the image cross‐sections (Figure 4h,i) along the root (blue dotted line in Figure 4a). In the OCT cross‐section (Figure 4h), the fine root hairs were visible, and exhibited very high LIV (Figure 4i), which may be because of their buoyancy in the covering water.

As shown in the inset of Figure 4h, individual cells were visible beneath the root hairs. In the superficial cell layers (red arrowhead), the cell structures were clearly visible. In contrast, in the deeper cell layers (green arrowhead), the cells were smaller, and the vertical cell walls were barely visible.

Similar to what was found in the en face LIV projections (Figure 4c,e), the hypocotyl had a high LIV in the cross‐section (Figure 4i). In addition, the root tip exhibited a very high LIV, which may indicate high growth activity in this region. It is also noteworthy that the adjacent tissue to the right of the root tip exhibited a low LIV.

**FIGURE 5:**
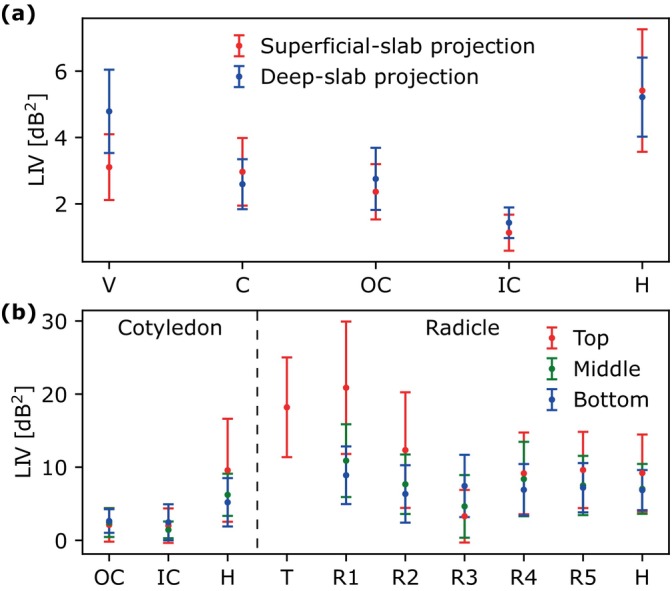
Mean and standard deviation values for the ROIs within inner (IC) and outer (OC) cotyledons (C), vein (V), radicle (R), radicle tip (T), and hypocotyl (H) of LIV images measured from Sample 1 on Day 2 (Figure 4). The plots correspond to (a) LIV slab projections at two depths (Figure 4c,e) and (b) LIV cross‐sections of cotyledon and radicle (Figure 4g,i).

The mean and standard deviation values for the ROIs within the vein (V), outer (OC) and inner (IC) cotyledons (C), radicle (T and R1–5) and hypocotyl (H) of LIV images are shown in Figure 5. The hypocotyl had higher LIV than the cotyledons (C, OC, and IC) and vein in both slab projections and the cross‐sections. The LIV of the vein (V) was higher than that of the region of the cotyledon (C) near the vein in the deep‐slab projection, but the LIV values of these tissues were close in the superficial‐slab projection. The outer (OC) and inner (IC) cotyledons far from the vein had higher LIV values in the deep region of the slab projection and the cross‐section, whereas the hypocotyl (H) had higher LIV in the superficial region. The LIV values of the radicle were calculated from 5 locations (R1–5) along the length from the tip toward the hypocotyl. The LIV of the tip (T) was higher than that of hypocotyl (H). The LIV in the radicle was higher on the side of the tip. Most of the regions had high LIV on the superficial (top) of the radicle.

#### 3.1.4 Days 5 and 11: Vessel Activity in Cotyledon and Hypocotyl

**FIGURE 6:**
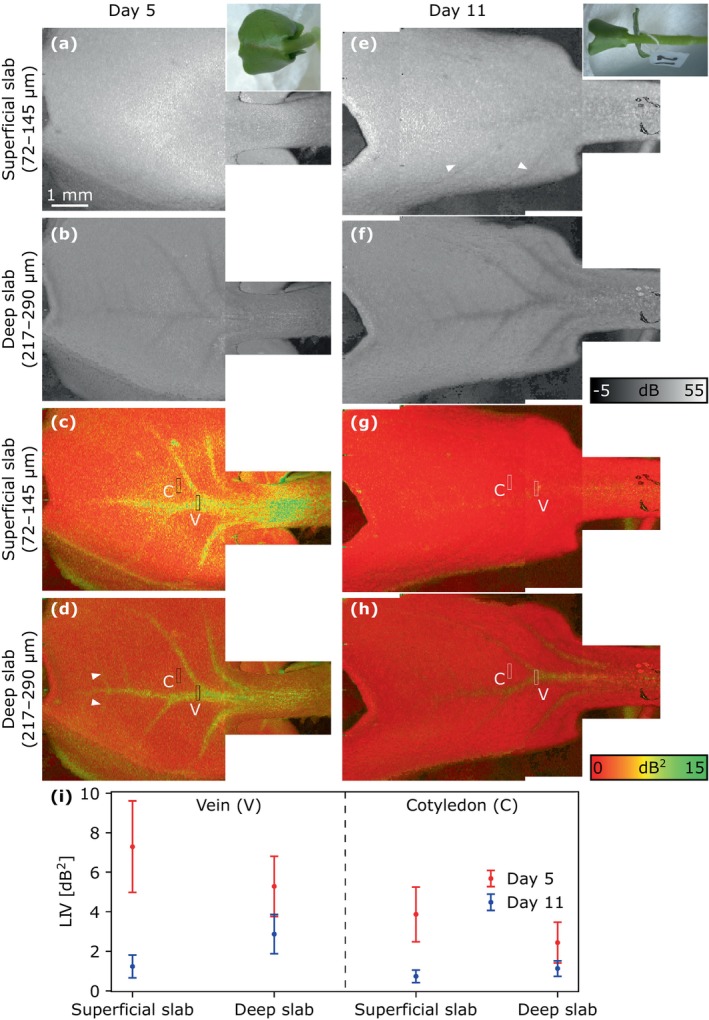
OCT intensity (grayscale) and LIV (pseudo‐color) images of the cotyledon of Sample 1 on Day 5 (left) and Day 11 (right). (a, e) The first and (c, g) third rows show superficial‐slab projections and (b, f) the second and (d, h) fourth rows show deep‐slab projections. On Day 5, the vessels exhibited a high LIV and low OCT intensity. On Day 11, the vessels were clearly visible as a low‐scattering structure in the OCT images but appeared dim in the LIV images. White arrowheads indicate fine vessel structure. The color photographs in the insets are reproduced from Figure 2. (i) The plot shows the mean and standard deviation values for the ROIs in the cotyledon (C) and vein (V) of LIV images.

Figure 6 summarizes the en face projections of the OCT and LIV images acquired on Days 5 and 11. ROIs were selected from the LIV images to calculate the mean and standard deviation values of the cotyledon and vein (Figure 6i). On Day 5, the en face deep‐slab projection (Figure 6d) of the LIV image clearly exhibited the vessel structure of a cotyledon, including fine vessels (white arrowheads) because the LIV in the vein was higher than in the cotyledon (Figure 6i). In the corresponding OCT intensity image (Figure 6b), the vessels exhibited hypo‐scattering. Notably, the superficial‐slab projection of the LIV image also showed a clear high‐LIV vessel structure on Day 5 (Figure 6c), whereas it had not been visible in the superficial‐slab projection on Day 2 (Figure 4c). In addition, the corresponding OCT image projection on Day 5 did not show such structure.

In contrast to the observations on Day 5, the vascular structure in the deep‐slab LIV image projection (Figure 6h) was dim on Day 11 and nearly invisible in the superficial‐slab LIV projection (Figure 6g). The cotyledon and vein had lower LIV values on Day 11 (Figure 6i), and this may be an indication of a low vessel activity or function in the cotyledon. Conversely, the hypo‐scattering vessel patterns in the deep‐slab OCT projection on Day 11 were clearer than those on Day 5 (Figure 6f). The superficial‐slab projection of the OCT image (Figure 6e) also exhibited vessel‐like structures, but their spatial patterns were not correlated with those in the deep‐slab OCT image projection. In addition, the LIV values in both the vein and cotyledon were higher in the superficial slab on Day 5 but higher in the deep slab on Day 11 (Figure 6i).

**FIGURE 7:**
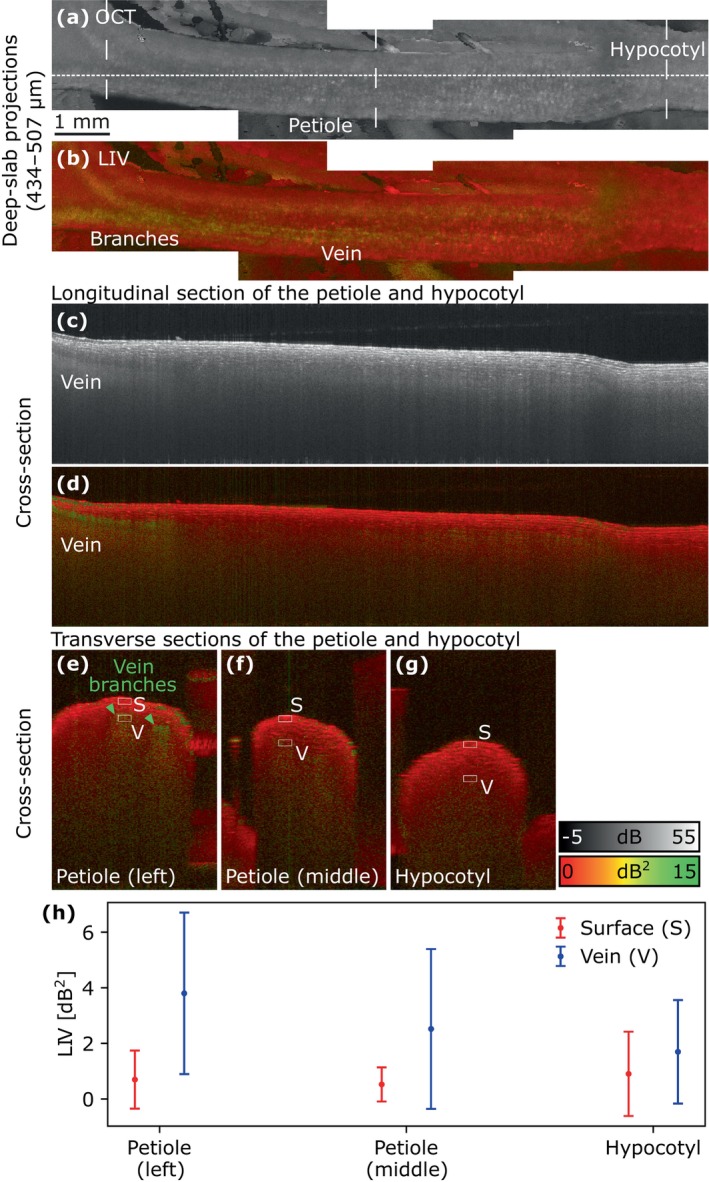
OCT intensity (grayscale) and LIV (pseudo‐color) images of the petiole and hypocotyl of Sample 1 on Day 11. (a, b) En face slab projections at a depth of 434–507 μm below the surface, and (c, d) longitudinal and (e–g) transverse sections. (h) Mean and standard deviation values for the ROIs within the epidermis (E) and vein (V) of LIV images (e–g). The dotted horizontal line in panel (a) indicates the location of the longitudinal section, and the dashed vertical lines correspond to the locations of the three LIV transverse sections. Green arrowheads indicate the vein branches.

The wide‐field composite images of the hypocotyl and petiole of Day 11 (Figure 7) enabled the visualization of vessel activities in these tissues. In the wide‐field deep‐slab LIV projection (Figure 7b), a vein running along the hypocotyl and petiole was visible. On the distal side of the petiole (left in the image), branching of the vein was observed, with a high LIV on the distal side (left) and a low LIV on the proximal side (right). In the cross‐section along the hypocotyl–petiole axis (Figure 7d) the vein had a high LIV on the distal side and low LIV on the proximal side. The same appearance was found in the transverse cross‐sections (Figure 7e–g).

The mean and standard deviation values of LIV were calculated from the transverse cross‐sections in Figure 7h. The surface was chosen to represent the tissue surrounding the vein. The vein exhibited relatively higher LIV values with larger variations than the surface, and its LIV decreases in the direction from the petiole toward the hypocotyl.

Notably, the high‐LIV vein exhibited hypo‐scattering in the corresponding OCT image (Figure 7c). This longitudinal OCT cross‐section also exhibited a horizontal stripe pattern between the vein and the surface of the petiole and hypocotyl. Despite the inability to see individual cells, the stripe pattern implies that the cells are sufficiently large for OCT to distinguish cell layers.

### 3.2 Functional Imaging of Root Growth

#### 3.2.1 Primary Root Growth

**FIGURE 8:**
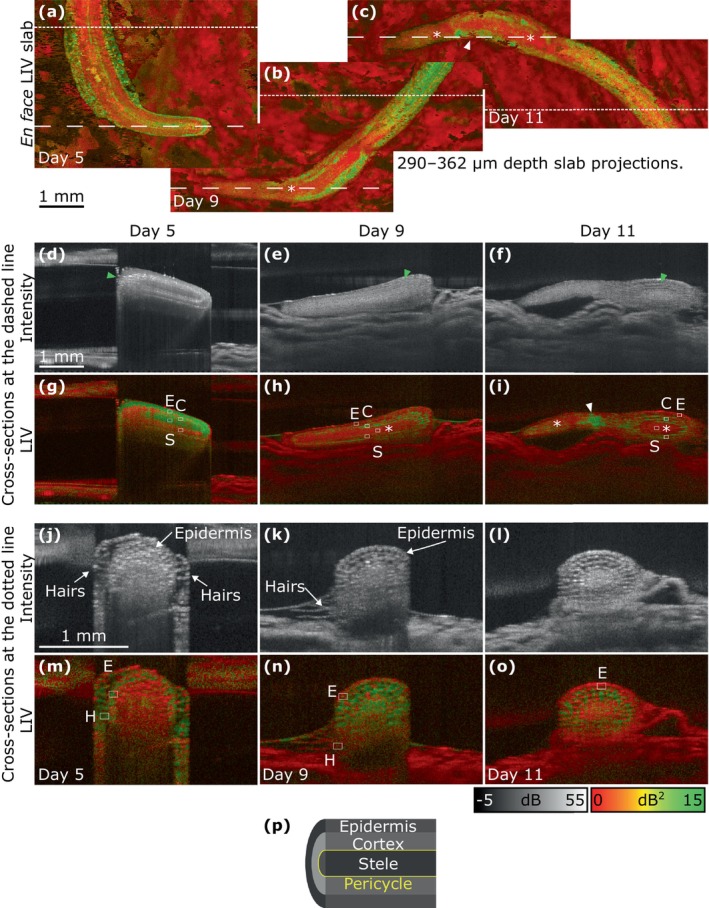
OCT intensity (grayscale) and LIV (pseudo‐color) images of the root of Sample 1 on Days 5, 9, and 11. (a–c) En face slab LIV projections (290–362 μm below the surface). (d–i) Longitudinal sections. (j–o) Transverse sections. (p) Schematic diagram of root anatomy. Dashed and dotted lines in (a–c) indicate the locations of the longitudinal sections and magnified transverse sections, respectively. Asterisks in (b, c, h, and i) indicate the stele. White arrowheads in (c, i) indicate the cortex. Green arrowheads in (d–f) indicate the large cortical cells.

Figure 8 shows the OCT intensity and LIV of the root measured on Days 5, 9, and 11, as well as a schematic diagram of root anatomy, including the epidermis, cortex, pericycle, and stele. Except the pericycle, these tissues were visible on Day 5 in Figure 8a,d,g,j,m. The cross‐sectional images of the epidermis, which is the outermost part of the root revealed a relatively low OCT intensity (Figure 8d) and high LIV (Figure 8g). The cortex, which is the layer beneath the epidermis, showed a high‐intensity lamellar structure in the OCT intensity cross‐section (Figure 8d) and a mixture of high and low LIV signals in the LIV cross‐section (Figure 8g). The stele, which is the central part of the root, had a low intensity in the cross‐sections (Figure 8d,j). In the slab projection (Figure 8a) and cross‐section (Figure 8g), the stele had a high LIV.

On Days 9 and 11, the cross‐sectional OCT intensity images (Figure 8e,f) exhibited a homogeneous appearance around the root tip, with the epidermis, cortex, and stele having indistinguishable intensities. In contrast, these tissues were distinguishable in the LIV images (Figure 8b,c,h,i,o). Moreover, the stele had a low LIV around the root tip on Days 9 and 11 (indicated by asterisks in Figure 8b,c,h,i), which contrasts with the high LIV seen on Day 5. Notably, the high‐LIV regions indicated by white arrows in Figure 8c,i were not part of the stele but rather the cortex. At all timepoints, cortical cells located far from the root tip appeared to be large in the OCT intensity cross‐section (green arrowheads in Figure 8d–f).

As shown in the magnified transverse cross‐sections (Figure 8j–o), the epidermal and cortical cells were large enough to be resolved in the OCT intensity cross‐sections at all time points. However, the stele had a homogeneous OCT intensity (Figure 8j–l), suggesting that the cells in the stele were too small to be resolved. On Days 5 and 9, the root hairs were visible (Figure 8j,k). Both epidermis and hairs had a high LIV on Day 5 and a low LIV on Days 9 and 11 (Figure 8m–o).

**FIGURE 9:**
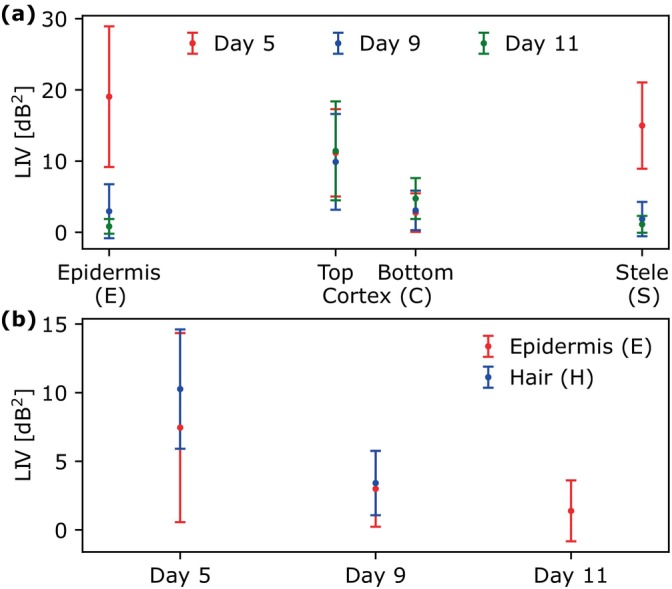
(a, b) Mean and standard deviation values of Days 5, 9, and 11 for the ROIs within the epidermis (E), cortex (C), stele (S), and root hair (H) of the LIV images in Figure 8.

To further examine the root tissues, the LIV values were plotted in Figure 9. The epidermis and stele had higher LIV values on Day 5 than that on Days 9 and 11 (Figure 9a). The LIV of the cortex measured from the bottom of the root was lower than that measured from the top on Days 5, 9, and 11. In the cross‐sections of the root on Days 5, 9, and 11 (Figure 8m–o), the root hair had higher LIV values than that of the epidermis, and the LIV of both tissues decreased over time (Figure 9b). Note that the ROI was not selected for root hair on Day 11 due to the inability to identify it from the OCT intensity and LIV cross‐sections.

#### 3.2.2 Lateral Root Growth

**FIGURE 10:**
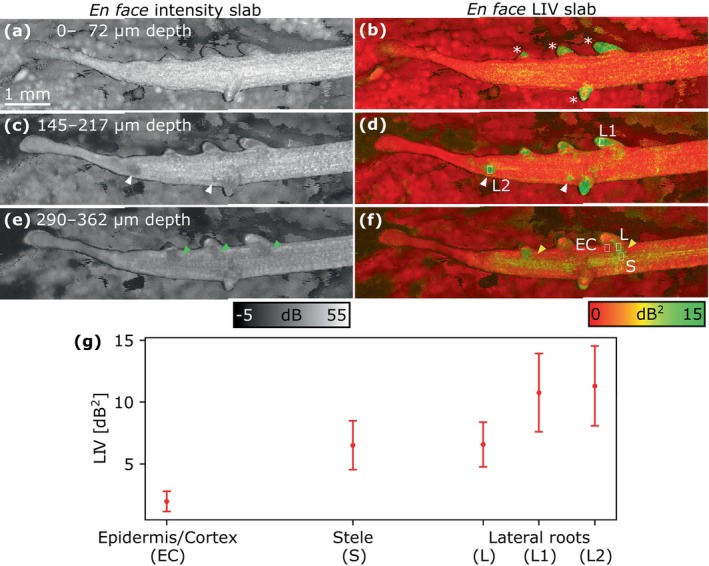
En face slab projections of OCT intensity (grayscale) and LIV (pseudo‐color) of Sample 2 on Day 9, showing lateral roots branching from the primary root. Slabs were obtained at depths of (a, b) 0–72, (c, d) 145–217, and (e, f) 290–362 μm below the surface. (g) Mean and standard deviation values for the ROIs within the epidermis/cortex (EC), stele (S), and lateral roots (L, L1, and L2) of the LIV slabs. Asterisks and white arrows indicate the emerged and emerging lateral roots, respectively, the latter not being visible from the surface. Green arrows indicate the lateral roots with low OCT intensity. Yellow arrows indicate the connections between the lateral roots and the stele.

The en face slab projections of the OCT intensity and LIV are shown for Sample 2 on Day 9 at three depths in Figure 10. The mean and standard deviation values of LIV were calculated from the ROIs in the epidermis, cortex, and stele of the root and the lateral roots of the projections (Figure 10g). Since the epidermis and cortex were indistinguishable using OCT intensity and LIV, an ROI outside the stele was chosen to calculate a LIV value representing both the epidermis and cortex, referred to as EC.

The primary root and its tip appeared as a low‐LIV structure, and the lateral roots appeared as high‐LIV structures, as indicated by the asterisks in Figure 10b. In a deeper slab, two newly emerged lateral roots were visible as structures with low OCT intensity and high LIV, as indicated by the white arrowheads in Figure 10c,d. In the deepest slab (Figure 10e,f), the lateral roots and the stele were connected by a structure with high LIV and low intensity as indicated by the yellow arrowheads in the LIV image and green arrowheads in the OCT intensity image. The stele (S) and lateral roots (L, L1, and L2) had higher LIV compared to the epidermis and cortex (EC) (Figure 10g). Notably, the emerged (L1) and newly emerged (L2) lateral roots exhibited even higher LIV values. The stele was more clearly identifiable in the LIV image (f) than in the intensity image (e).

## 4 Discussion

### 4.1 Visualization of Plant Growth and Tissue Maturation

#### 4.1.1 Growth Rate and LIV

All newly emerged radicles and hypocotyls grew faster than the cotyledons on Days 1 and 2 as shown in the photographs (Figure 2a–c) and OCT images (Figures 3 and 4), with fast‐growing structures exhibiting a high LIV.

As a specific example, the cotyledon showed a high LIV on Day 2 (Figure 4) and Day 5 (Figure 6), whereas it showed only a low LIV on Day 1 (Figure 3) and Day 11 (Figure 6). This suggests that the LIV is a measure of the growth rate of the tissue.

#### 4.1.2 Visualization of Vascular Development and Maturation

Procambium, which is a meristematic tissue, is known to form the vasculature of cotyledons during embryogenesis and differentiates into xylem and phloem during germination [35]. Therefore, it is reasonable to assume that the cotyledons already had vasculature on Day 2, and thus the high‐LIV vein structure observed at this time (Figure 4e) may correlate with the differentiation of procambium.

The differentiated vascular cells eventually mature during the development of cotyledons. The relatively low LIV on Day 11 (Figure 6g,h) might indicate the low growth rate associated with maturation of the vascular cells.

### 4.2 Substance Transportation in Vasculature

The xylem and phloem are known to transport water and nutrients, respectively, and laser speckle imaging has successfully visualized the water transport in leaves by analyzing the temporal fluctuation of speckle patterns [31, 32]. The fluctuation is believed to result from the particle movements of transporting substances. Considering the close proximity of the xylem and phloem, the speckle fluctuation may originate from both water and nutrient transport.

Similar to laser speckle imaging, D‐OCT also relies on the temporal fluctuation analysis of speckle. Hence, it is likely that D‐OCT is also sensitive to water and nutrient transport. In addition, as discussed in the previous section, D‐OCT can also be sensitive to tissue activities associated with growth, such as cellular division.

On Day 2 (Figure 4c,e) and Day 5 (Figure 6c,d), the hypocotyl and veins in the cotyledon both exhibited a high LIV. By Day 11, the matured veins in the cotyledon (Figure 6h) and petiole (Figure 7b,d) showed a high LIV, while the hypocotyl showed a low LIV. These findings are consistent with our hypothesis that D‐OCT is sensitive to both water/nutrient transport and cellular activity associated with tissue growth.

### 4.3 Variation Among Sprouts

For all seven radish samples, the radicle, cotyledon, and hypocotyl tissues were found to exhibit similar LIV appearances on Days 1 and 2. Changes in the LIV of the cotyledons and veins followed a similar pattern across all plants over the 11‐day measurement period. D‐OCT measurements were highly reproducible across individual plants.

The morphology and LIV changed as the sprouts grew. For instance, the locations and areas of high LIV in the root cortex of Sample 1 were different on Days 5 and 11, as shown in Figure 8. In addition, temporal changes in the morphology and LIV patterns also differed among individual sprouts. Near the root tip, Sample 2 had lateral branches on Day 9 (Figure 10), whereas Sample 1 did not form any branches between Days 5 and 11 (Figure 8). Among the seven sprouts, two developed lateral branches within 10 mm from the root tip, and one of them was Sample 2.

Environmental factors such as gravity, water availability, and competition among individual sprouts can cause variations in root development [36, 37, 38]. Since the sprouts were grown in a petri dish, competition among the roots was unavoidable. Each sprout was moved and placed in another petri dish prior to OCT measurement, and this movement could have caused changes in gravity and water availability that resulted in variations among the sprouts.

### 4.4 Morphology and Cellular Activity of Roots

#### 4.4.1 Roots in Early Development

As radish seeds undergo epigeal germination, the radicle elongates to break through the seed coat, the hypocotyl elongates, and then cell division occurs at the root tip [39]. Our OCT findings on Days 1 and 2 (Figures 3 and 4) are consistent with these events.

**FIGURE 11:**
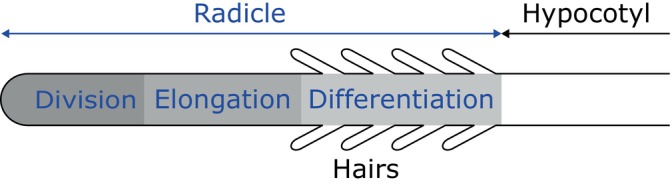
Diagram of cellular processes in the radicle.

The cellular processes along the length of the emerged radicle can be divided into zones of division, elongation, and differentiation [40] as shown in Figure 11. Specifically, the root hairs are found in the differentiation zone, which may explain their high LIV in Figure 4i. The elongation zone is known to have large cells without root hairs [36]. Consistent with this, the elongation zone with large cells (yellow arrowhead in Figure 4h) and hypocotyl, which flank the differentiation zone, do not have root hairs.

### 4.5 Lateral Roots and Surrounding Tissues

Lateral roots are known to originate from the pericycle (the outermost layer of the stele shown in Figure 8p) and emerge through the endodermis, cortex, and epidermis [41]. In the LIV image of Sample 2 (Figure 10f), the lateral roots appeared as high‐LIV structures originating from the stele and emerging through the low‐LIV layer. Although the endodermis, cortex, and epidermis were indistinguishable in both the OCT intensity and LIV images of the lateral roots (Figure 10e,f), these tissues collectively appeared as a low‐LIV layer.

Emerging lateral roots grow faster than the surrounding tissues of the primary root. This fast growth may be associated with organelle motility driven by high metabolism and thus be the cause of the high LIV of the lateral roots.

### 4.6 D‐OCT for Plant Imaging

#### 4.6.1 Comparison With Fluorescence Confocal Microscopy

Fluorescence confocal microscopy is a widely used plant imaging technique that provides higher contrast and resolution than OCT. By labeling specific molecules with fluorophores, it produces a bright image against a dark background. In contrast, OCT is prone to speckle noise, which reduces contrast and often sacrifices resolution to achieve greater imaging depth.

While OCT offers greater imaging depth, it also has the advantage of faster volumetric data acquisition. D‐OCT, as a label‐free imaging technique, eliminates the need for potentially harmful fluorophores, making it a safer alternative for plant imaging.

#### 4.6.2 Limitations and Solutions

The penetration depth of D‐OCT is limited to approximately 1 mm in plant tissues due to light attenuation, as observed in LIV cross‐sections. The attenuation reduces the reliability of LIV measurements in deeper regions, which appear dark in LIV images due to low OCT intensity. Consequently, D‐OCT is not suitable for measuring the thick tissues of grown plants. While imaging both sides of a thicker plant could be considered, this approach is impractical for very thick specimens.

We acknowledge that LIV is unable to differentiate between various cellular processes such as cell division, elongation, and substance transport. However, analyzing the fluctuation rate or frequency of OCT signals may help distinguish between these processes.

The speed of cellular and tissue alteration during the cellular processes may slow down as the plant grows. Our current protocol traced the plant growth only up to 11 days, and further longer longitudinal imaging is expected to thoroughly examine these extended processes.

As our results implied, root growth is highly affected by the environmental factors, such as water availability and sample movement. We believe that growing and measuring the plants within a controlled environment may effectively reduce root variability.

### 4.7 Future Impacts

The results demonstrated the capability of LIV to visualize growth‐related activities within the deep tissues of living plants at various developmental stages. These visualizations provide valuable insights into the timing and spatial distribution of these activities across different organs, potentially advancing plant research.

Notably, D‐OCT enables the non‐invasive acquisition of internal information from relatively thick plant tissue, eliminating the need for physical contact that could disrupt plant growth. Since sample preparation is not required, D‐OCT can be applied to a broader range of plant species compared to traditional methods like microscopy.

In agriculture, LIV may serve as a growth metric for selecting fast‐growing seeds and sprouts, thereby enhancing overall production. Additionally, this growth metric could be used to optimize the supply of resources such as water and nutrients.

## 5 Conclusion

The cotyledons, hypocotyls, and roots of germinated radishes were measured using D‐OCT for 12 days. The results suggest that D‐OCT can visualize water and nutrient transport and also cellular activities associated with tissue growth. Since D‐OCT is a three‐dimensional and non‐invasive imaging modality, it may enable volumetric, longitudinal in vivo imaging of plant development. D‐OCT may therefore become a valuable tool in plant biology.

## Author Contributions

Y.L., S.K., and Y.Y. equally contributed to this study. Y.L. and Y.Y. were involved in conceptualization, investigation, writing – original draft, and project management. S.K. was involved in conceptualization and investigation. P.M., I.A.E.‐S., and S.M. were involved in the measurement methodology development and writing – review and editing.

## Conflicts of Interest

All authors have received research funding from Sky Technology, Nikon, Kao Corp., Topcon, Panasonic, and Santec.

## Data Availability

The data that support the findings of this study are available from the corresponding author upon reasonable request.
